# Rubber Patents

**Published:** 1881-06

**Authors:** 


*Rubber Patents.*—Although the Cummings' patent, owned by the Goodyear Dental Vulcanite Company, expires June 7th, and the company has as yet made no effort to obtain from Congress an extension of that patent, it seems it does not follow that dentists are safely free from that monopoly. According to present patent laws, application for extension need not necessarily be made on or before the expiration of a patent, but may be made at a subsequent Congress. Then, too, it would seem that this company own the Hæring patent, which, curious as it may seem, is almost an exact equivalent of the Cummings', and it seems more than probable that dentists may be compelled to work under licenses issued by the owners of this patent, just as before. It may be well, then, not to be hasty in congratulating ourselves on an escape as a profession from the grasp of a monopoly, for if the Goodyear Dental Vulcanite Company can, they assuredly will continue their license system. H.

